# Novel Insights on Intake of Fish and Prevention of Sarcopenia: All Reasons for an Adequate Consumption

**DOI:** 10.3390/nu12020307

**Published:** 2020-01-24

**Authors:** Mariangela Rondanelli, Chiara Rigon, Simone Perna, Clara Gasparri, Giancarlo Iannello, Rashida Akber, Tariq A. Alalwan, Afnan Mahmood Freije

**Affiliations:** 1 IRCCS Mondino Foundation, 27100 Pavia, Italy; mariangela.rondanelli@unipv.it; 2 Department of Public Health, Experimental and Forensic Medicine, University of Pavia, 27100 Pavia, Italy; 3 Endocrinology and Nutrition Unit, Azienda di Servizi alla Persona Istituto Santa Margherita, University of Pavia, 27100 Pavia, Italy; chiara.rigon01@universitadipavia.it; 4 Department of Biology, College of Science, University of Bahrain, Sakhir Campus, P.O. Box 32038, Zallaq, Bahrain; sperna@uob.edu.bh (S.P.); rashida_akber@hotmail.com (R.A.); talalwan@uob.edu.bh (T.A.A.); afreije@uob.edu.bh (A.M.F.); 5 General Management, Azienda di Servizi alla Persona Istituto Santa Margherita, 27100 Pavia, Italy; direttoregenerale@asppavia.it

**Keywords:** sarcopenia, fish, muscle mass, anti-inflammatory

## Abstract

Sarcopenia is defined as a syndrome characterized by progressive and generalized loss of skeletal muscle mass and strength and it is diagnosed by measurements of muscle mass, muscle strength, and physical performance. Sarcopenia affects quality of life and is associated with several adverse health effects. Muscle decline is aggravated by a sedentary lifestyle and can be prevented through proper nutrition, together with adequate physical activity. Fish contains biologically active compounds, such as omega-3 polyunsaturated fatty acids, proteins, vitamin D, magnesium, and carnitine, which are able to intervene positively on muscle metabolism. This narrative literature review was performed to evaluate evidence regarding the actual benefit of fish consumption in the prevention of sarcopenia and the positive action on the muscle mass of the biological compounds present in fish. The results demonstrated that fish consumption has a protective and anti-inflammatory function on skeletal muscle and that its biologically active compounds help to maintain good muscle performance, preventing sarcopenia. Considering the nutritional and health benefits, elderly with sarcopenia should consume at least three servings per week of fish in order to have a minimum intake of 4–4.59 g daily of omega 3, and reaching the 50% RDA in Vitamin E and D. High biological value of proteins in 150 g of fish and its high available magnesium (20% of RDA in 150 g of fish) are an added value that could suggest fish as a “functional food” in order to prevent and treat sarcopenia.

## 1. Introduction

Sarcopenia has been defined by the European Working Group on Sarcopenia in Older People (EWGSOP) as a syndrome characterized by progressive and generalized loss of skeletal muscle mass and strength with a risk of adverse outcomes such as physical disability, poor quality of life, and death [1]. The magnitude of decline in fat free mass (FFM) is greater in men than in women and is amplified in sedentary individuals relative to their physically active peers [2]. The aged muscle has a reduced ability to up-regulate protein synthesis in response to anabolic stimuli, such as protein intake and exercise [3], and insulin and insulin-like growth factor I (IGF-I) [4]. This reduced sensitivity to anabolic stimuli termed “anabolic resistance” leads to a negative net protein balance causing losses of skeletal muscle protein and results in sarcopenia [5].

Nutrition plays an important role in the development of sarcopenia as food intake declines gradually throughout adulthood. Energy intake drops by about 600 kcal in women and 1330 kcal in men between the ages of 20 and 80 years [6].

Epidemiological data indicate that older persons are at a higher risk for inadequate protein intake than their younger counterparts [7]. For this reason, the recommended amount of protein intake is 1.2 g/kg/day for healthy older adults [8], 1.5–2.0 g/kg/day for those who have acute or chronic disease, and rises up to 2.0 g/kg/day during periods of acute illness [5,9].

In addition to proteins, many other nutrients are essential for the maintenance of muscle mass, such as essential amino acids, amino acid metabolites, creatine monohydrate, antioxidants, omega-3 polyunsaturated fatty acids (PUFAs), vitamin D, vitamin B6, folic acid, and magnesium [10,11]. Other new experimental compounds have also been identified, such as caloric restriction mimetics and manipulation of the gut microbiota [11]. In recent years it has become increasingly evident that the gut microbiota has a strong influence on the health of the individual, in particular, fish consumption has not only demonstrated protective effects against heart disease, metabolic diseases, and even some cancers [12], but also on the integrity of the intestinal microbiota: pregnant women who meet the recommended fish consumption (2–3 times a week) are more likely to have a dominant Bifidobacterium profile than a dominant Escherichia profile [13]. Studies in recent years reveal that the intestinal microbiota also plays a decisive role in relation to muscle; in fact, during old age, the bacteria that produce butyrate (which influence colon motility, immunity maintenance, and anti-inflammatory properties) decrease, while the bacteria belonging to Proteobacteria phylum increase [14,15,16]. This dysbiosis causes greater intestinal permeability promoting the passage of inflammatory molecules such as LPS (lipopolysaccharides), which are very high in the elderly. This endotoxin leads to systemic inflammation which affects the skeletal muscle, affecting its integrity [17].

The current recommendation for the consumption of fish (at least twice a week) [18] is important for human health [19] as it protects against the risk of developing cardiovascular disease (CVD) [20], type 2 diabetes (T2D) [21], and possibly some cancers [22]. Moreover, it contains nutrients that may be beneficial in the prevention of sarcopenia [23].

Although fish contains many nutrients that are potentially beneficial for maintaining muscle health, very few studies in the literature evaluate the effect of fish consumption and its nutrients on maintaining muscle mass. The aim of the present narrative review is to summarize the state of art regarding the effect of preventative intake of active compounds in fish, such as omega-3 PUFAs, proteins, vitamins D and E, magnesium, and carnitine in sarcopenia.

## 2. Omega-3 Polyunsaturated Fatty Acids Effects on Skeletal Muscle Mass

The mechanism through which sarcopenia manifests itself is not fully known, but muscular stem cells, also called “satellite cells,” play an important role. They are responsible for skeletal muscle repair following damage [24] and have been implicated in regulating extracellular matrix production during hypertrophy and regenerative processes, and therefore play an important role in the maintenance of muscle mass with advancing age [25,26].

We have considered works since 2007 in which the authors focused on the muscle-level effect of omega-3 polyunsaturated fatty acids supplementation. We also included the only work in which no supplementation was given but the effect of consumption of fish on grip strength was examined.

Several evidences indicate that omega-3 PUFAs are able to reduce muscle wasting by increasing the functional capacity in the elderly by growing the intracellular metabolic signal [27,28]. The most common omega-3 PUFAs in the diet are α-linolenic acid (ALA) present in plants and seeds, and docosahexaenoic acid (DHA) and eicosapentaenoic acid (EPA) that are both present especially in cold water fish species such as salmon and tuna [28]. Another essential fatty acid found is linoleic acid (LA) which is part of the omega-6 PUFAs. ALA and LA are essential in the human diet since they are indispensable constituents of cell membranes and cannot be synthesized from other fatty acids, while EPA and DHA can be synthesized in the endoplasmic reticulum in liver cells through conversion from ALA, though this conversion is limited in the human body due to enzyme availability [29,30].

EPA and DHA act as substrates for the synthesis of lipid-derived mediators of inflammation. These mediators, termed eicosanoids, vary in their ability to mediate inflammation depending on the substrate used for their synthesis. For example, eicosanoids derived from arachidonic acid, an omega-6 PUFA, are considered to be pro-inflammatory [28].

The typical Western diet is deficient in omega-3 PUFAs and abundant in omega-6 PUFAs [31]. A high omega-6/omega-3 ratio is linked to an increased state of chronic inflammation, which has been linked to diseases such as T2D and obesity [31]. Increased levels of inflammation in turn are associated with an increased production of pro-inflammatory cytokines. In the elderly, a high presence of pro-inflammatory cytokines prevents proper muscle regeneration by impairing satellite cell differentiation and fusion and by increasing the activation of nuclear factor-KB (NF-KB) transcription factors, thereby contributing to the deterioration of muscle mass with age [28]. The omega-3 PUFAs (DHA and EPA) are known for their anti-inflammatory properties [32], and marine-derived omega-3 PUFAs can influence the exercise and nutritional response of skeletal muscle [33].

Few studies have been carried out regarding the effects of omega-3 PUFAs on muscle cells and, apart from McGlory [34] and Pahor [35] who reported no effects, the other studies have identified changes after omega 3-PUFAs supplementation. For example, Smith et al. [27] demonstrated that supplementation with long-chain omega-3 PUFAs of 4 g/day (1.86 g EPA and 1.5 g DHA) for six months reduced the normal decline in muscle mass and function in healthy older adults (60–85 years) in comparison with the corn-oil supplemented placebo group. The authors reported that treatment with fish oil-derived omega-3 PUFAs had increased thigh muscle volume, handgrip strength, and the average isokinetic leg muscle power [27]. Moreover, expanding upon Smith and colleagues initial work, Yoshino and Smith [36] selected ten subjects who had responded best to the treatment group and ten subjects from the control group who matched with the chosen ones of the treatment group for age, sex, body mass index (BMI), and overall compliance to the protocol. Using the microarray technique, they found that different pathways were involved in mitochondrial regulation and in the organization of the increased cell matrix while the pathways related to calpain- and ubiquitin-mediated proteolysis, mRNA translation, and inhibition of the mammalian target of rapamycin (mTOR) kinase signaling were decreased by omega-3 PUFAs therapy. Such effects may help explain the anabolic and function enhancing effects of omega-3 PUFAs therapy [36].

Several clinical studies also investigated the effect of omega-3 PUFAs on both genders. In a study involving elderly women, Rodacki [37] showed that supplementation with fish oil accompanied by strength training improved the response of the neuromuscular system and enhanced their muscle strength and functional capacity [37]. Interestingly, Da Boit et al. [38] discovered a different reaction of muscle mass to omega-3 PUFAs supplementation in females compared to males. In their study, the authors recruited 50 patients from both genders, and they were divided into two randomized groups. Group 1 received 3 g of fish oil and group 2 received a placebo. The investigators showed that long-chain omega-3 PUFAs supplementation had increased maximal isometric torque and muscle quality values after 18 weeks of resistance exercise in elderly females, but not male patients [38].

The changes in the ratio between muscle protein synthesis (MPS) and muscle protein breakdown (MPB) regulate muscle protein balance. The balance is positive (i.e., muscular hypertrophy) when MPS increases or MPB decreases. When the muscle is not used due to injury or illness, there is a rapid loss of skeletal muscle mass [39]. For instance, Smith et al. [40] showed that eight weeks of omega-3 PUFAs supplementation enhanced hyperinsulinemic-hyperaminoacidemic induced increases in MPS in elderly adults. Further, Mcglory et al. [34] demonstrated that eight weeks of fish oil supplementation (4.5 g/day omega-3 PUFAs) in young, healthy males had no effect on MPS measured after resistance-based exercise and the ingestion of 30 g of whey protein.

Animal and in vitro studies have also been conducted in an effort to elucidate the effects of omega-3 PUFAs on skeletal muscle health. For instance, Kamolrat et al. [31] reported that EPA, but not DHA, significantly enhanced protein synthesis and reduced protein breakdown in murine C2C12 myotubes.

Furthermore, Gingras et al. [41] reported that fish oil feeding enriched in menhaden oil to growing steers increased whole body protein synthesis via the activation of the Akt-mTOR-p70s6k anabolic signaling pathway. The researchers showed that long-term enteral of the supply of long chain omega 3-PUFAs gives greater sensitivity to elimination of insulin-regulated amino acids and glucose and that these results occur, in part, in the skeleton muscle. Similarly, in an epidemiological study conducted among 2983 older adults (59–73 years), Robinson et al. [42] reported that fatty fish consumption was the most influential dietary component positively affecting grip strength. The results of these studies, summarized in Table 1, are contradictory, with the majority reporting that omega-3 PUFAs may be useful for the prevention of sarcopenia. In the two studies that showed no effect of omega-3 PUFAs, the duration of the study by Mcglory et al. [43] was short compared to other studies, while the study by Pahor [35] assessed long-term omega-3 supplementation on a large number of very compromised patients.

## 3. Vitamin E in Farmed Fish

Like many other PUFAs, EPA and DHA are prone to lipid peroxidation because of the presence of double bonds [44]. For this reason, in recent years, farmed fish, such as Atlantic salmon, has been enriched with vitamin E. Vitamin E is a known biological antioxidant and has been shown to inhibit the oxidation of long-chain PUFAs in oily fish [45]. The free radical-scavenging vitamin E comprises of four classes of tocopherols named according to the position and number of the methyl groups on the phenol ring, namely alpha, beta, gamma, and delta [46]. Levels of gamma tocopherol are inversely associated with cardiovascular disease risk in humans [47]. For this reason, Menoyo et al. [44] investigated the transfer of dietary gamma tocopherol (gT) into marine animal species by feeding farmed Atlantic salmon with gT-rich diets for 16 weeks. The authors reported a three-fold increase in gT concentrations in the liver and fillet compared to non-gT-supplemented controls. In addition, dietary gT had decreased malondialdehyde values by 25%, thereby demonstrating lower lipid peroxidation [44].

## 4. Fish Proteins Function on Muscle Mass

Skeletal muscle contractile proteins represent the largest protein reservoir that act in an anabolic fashion in nutrition and can be quickly used to supply amino acids to the whole body during fasting or stress. For this reason, adequate protein intake is of fundamental importance as a negative protein balance leads to atrophy of the skeletal muscles, reduced muscle growth, and a decline in functional status [48].

Epidemiological data indicate that the elderly are at high risk for inadequate protein intake. For women, 32%–41% and 22%–38% of men over the age of 50 consume less protein than recommended (RDA for proteins 0.8 g·kg^−1^·day^−1^) and virtually no older adult receives the highest Acceptable Macronutrient Distribution Range (AMDR) for protein (35% of total energy intake) [10].

The aged muscle has a reduced ability to up-regulate protein synthesis in response to anabolic stimuli, such as protein intake and exercise [3], but the muscle mass of older individuals retains the ability to mount a robust anabolic response following the ingestion of protein-rich meals [49]. In addition to consuming the recommended amount of protein (1.2 g/kg body weight/day), the distribution of dietary protein throughout the day is important in older adults [48].

The fast and slow muscle fibers have different metabolic characteristics and are arranged in a heterogeneous manner in the skeletal muscle. Muscular atrophy and hypertrophy mainly affect fast fibers [50].

Insulin resistance and inflammation play an important role in muscle mass decline because they are considered to be risk factors for the development of age-related loss of muscle mass and function [51]. Several studies have shown that adequate intake of proteins of animal or vegetable origin contributes to the maintenance of lean mass, but very few have focused on fish proteins [52,53].

In rats, fish proteins have been shown to influence blood lipid concentrations [54,55]. Furthermore, Kawabata et al. [56] reported that the consumption of fish proteins resulted in less epididymal adipose tissue and lower levels of plasma triglycerides in rats compared with the consumption of casein. In the same study, the authors found that fish protein was able to increase skeletal muscle weight, especially the fast-type muscle and fast-twitch fibers [56].

These results confirm those of a previous study [57], which indicated that feeding 5-week-old male Sprague-Dawley rats a high-fat diet with Alaska pollack protein (APP) for four weeks resulted in higher weight of gastrocnemius muscle, which is rich in fast-twitch fiber compared to casein-fed rats [57].

Similarly, a recent study using 5-week-old male Sprague-Dawley rats showed that the volume of gastrocnemius increased in the APP-fed rats in comparison to the casein-fed animals. The results indicated a volume increase in both fast and slow fibers [50].

Although human trials have not yet been conducted with fish protein supplementation alone, studies have been done to evaluate the effect of animal protein (including fish proteins) consumption on the lean mass. For example, the recent study by Bradlee et al. [58] evaluated the effect of animal and plant proteins with or without physical activity. The authors found that the intake of animal proteins was related to a greater lean mass while the benefits of vegetable proteins were only visible in those who carried out intense physical activity [58].

Another study by Alexandrov et al. [51], also using animal protein (i.e., fish, meat, and eggs), demonstrated that protein intake is able to contribute to maintaining and building muscle mass. Furthermore, they measured the creatinine produced by the subjects and from the results they concluded that animal-derived proteins may preserve muscle mass. However, they indicated that further research was needed to evaluate the effect of different dietary protein sources on muscle mass. Table 2 summarizes human studies.

## 5. Fish Consumption Increases Vitamin D Levels

Vitamin D is unique because it can be made by the body through exposure to sunlight [59]. Solar ultraviolet B photons are absorbed by 7-Dehydrocholesterol (provitamin D_3_, 7-DHC), which is synthesized inside the liver from cholesterol and converted to previtamin D3. Under the influence of heat, it immediately converts to vitamin D3 (cholecalciferol) [60]. Vitamin D is also ingested from the diet [61] as vitamin D2 (ergocalciferol), and is especially found in plants or plant products, while cholecalciferol is mainly contained in animal products like fresh and canned salmon, mackerel, and tuna, as well as cod liver oil [60,62], meat, eggs, and dairy [63]. Cholecalciferol is absorbed in the small intestine and then transported to the liver by a transport α-2 globulin vitamin D-binding protein for hydroxylation to 25-hydroxycholecalciferol (25OHD3). Subsequently, in the kidney, 25OHD3 is hydroxylated to form 1,25-dihydroxyvitamin D3 [1,25-(OH)2D3], the biologically active form of vitamin D3 [64].

Vitamin D is essential for bone metabolism (its deficiency increases the risk of fractures) [65] and low levels of vitamin D are associated with the increased risk of developing autoimmune, neurogenerative, and cancer diseases [61]. Vitamin D is also involved in neuromuscular function. In fact, it affects muscle strength [62], muscle size, and muscle performance [66]. The age-related decline in muscle mass and function is concurrent with a decline in skeletal muscle vitamin D receptor expression [67]. In fact, the active form of vitamin D binds to a highly specific nuclear receptor in the muscle tissue [64,68] and leads to protein synthesis and growth of muscle tissue [64]. Weekly fatty fish consumption significantly increases vitamin D levels [69]. A study carried out by Lu et al. [65] showed wild salmon as having the highest vitamin D3 content, while the farmed fish exhibited a 25% reduction in the content of vitamin D3.

An interesting study by Granic and co-workers [70] demonstrated that during periods of the year when sunshine is at a relative minimum and in the absence of vitamin D supplementation, there is an accelerated loss of muscle mass in elderly people. For this reason, it is important to include vitamin D-rich fish in the dietary pattern.

Few individuals consume fish frequently and stay outdoors for more than an hour a day, so vitamin D levels are often inadequate. We considered the works that evaluated a direct correlation between the levels of vitamin D and sarcopenia (without supplements) and we also included in the research a study that assessed whether a supplementation greater than 24,000 IU could be useful or harmful in people aged 70 years and older.

Results have shown that vitamin D is important for maintaining muscle mass. For instance, Kim et al. [71] reported the results of a cross-sectional analysis performed on 3169 older Korean adults. The results showed a strong inverse association between serum 25(OH)D and sarcopenia in the participants of the study. This association was independent of age, sex, BMI, occupation, and lifestyle factors such as alcohol consumption, smoking, and regular exercise [71]. The main function of vitamin D is to maintain calcium and phosphate homeostasis [64]. For this reason, the same authors of [71] evaluated the parathyroid hormone (PTH) levels of patients from the same study and reported that serum PTH was significantly increased in the elderly with sarcopenia.

In addition, Knutsen et al. [68] found a relationship between individuals with musculoskeletal pain, headache, fatigue, and vitamin D levels. In the multi-ethnic cross-sectional study of 572 patients, hypovitaminosis D were reported among 58% of the patients with the lowest levels found in those complaining of headaches [72].

Bischoff-Ferrari et al. [67] also reported an age-related decrease in vitamin D receptors. In their study, the authors examined gluteus medius biopsy samples from 20 elderly female patients (72% >65 years). Twelve patients were operated on for spinal fixation, decompression, or removal of prior fixation hardware. The authors found a significant decrease in vitamin D receptor expression with age in freshly removed muscle tissue of the female patients. Further, Snijder et al. [73] studied the participants of the Longitudinal Aging Study in Amsterdam and found that serum 25(OH)D levels less than 25 nmol/L were associated with high risk for falls in older individuals. Moreover, the active metabolite of vitamin D, 25(OH)D is essential in the regulation of calcium transport and protein synthesis in muscle cells, increasing the calcium pool which plays a crucial role in muscle contraction [74]. This does not mean, however, that high levels of vitamin D supplementation correspond to equally better muscle performance. For instance, Bischoff-Ferrari et al. [75] demonstrated in a randomized clinical trial that frail elderly patients who received 60,000 IU of supplementary vitamin D3 per month had increased risk of falls and fractures than other patients who were randomized to 24,000 IU per month after a 12-month follow-up period.

Vitamin D status may also influence fatty degeneration in muscle [71]. For example, in a study investigating potential rotator cuff injury, Oh et al. [64] found that serum 25(OH)D levels had a significant negative correlation with fatty degeneration of rotator cuff muscles and a positive correlation with isokinetic muscle performance in men and women with rotator cuff disorders. Furthermore, vitamin D is important for the maintenance and proper functioning of muscle mass (Table 3).

In this case, studies were chosen in which vitamin D supplementation was not given but the association between vitamin D levels and sarcopenia was assessed. In one case, work was considered in which integration was given to evaluate whether a high dose of supplementation of this hormone could be useful or harmful.

## 6. Central Role of Magnesium in Maintaining Muscle Mass

Magnesium is essential for the proper functioning of the human body. It has multiple physiological functions at the enzymatic and structural levels [76]. Some studies have shown that magnesium may play a role in insulin secretion in insulin-resistant patients, as the condition has been associated with hypomagnesemia in diabetic patients [77,78]. Magnesium is also involved in numerous enzymatic reactions in which it functions as a cofactor, stabilizing and activating many enzymes and many reactions that generate adenosine triphosphate (ATP), in addition to its crucial role in muscle relaxation and muscle function, normal neurological function, and release of neurotransmitters [79]. Magnesium, together with constant physical activity, is correlated also with an increase in testosterone levels. In athletes, magnesium supplementation is able to improve performance by increasing the plasma testosterone levels [80]. Muscle mass significantly associated with serum-free testosterone [81] and testosterone levels decrease at a rate of approximately 1% per year after the age of 30 [82]. Adequate levels of magnesium are therefore important at all ages but even more significant in old age.

Fish, above all, crustaceans and molluscs, are an excellent source of magnesium [83]. Magnesium plays an essential role in maintaining muscle mass. In athletes, magnesium depletion leads to structural damage of the muscle cells, possibly due to increased reactive oxygen species availability, lipid and proteins damage, and/or altered calcium homeostasis [84]. In the InCHIANTI study, Dominguez et al. [84] found a significant positive association of serum magnesium concentration with muscle performance in older adults. Similarly, in a cross-sectional study involving 2570 women aged 18 to 79 years, Welch et al. [85] observed a positive association of dietary magnesium with indices of skeletal muscle mass and leg explosive power, indicating that dietary magnesium may slow the age-related loss of skeletal muscle mass. Moreover, data from another prospective cohort study, conducted by Welch et al. [86], showed that a higher intake of magnesium was positively associated with greater grip strength and higher skeletal muscle mass. More recently, Hayhoe et al. [87] investigated the correlation between serum magnesium concentration and FFM. They analyzed 7-day food records from 14,340 middle- to older-aged men and women participating in the European Prospective Investigation into Cancer and Nutrition-Norfolk cohort study and estimated the correlation between FFM-based indices of muscle mass and dietary magnesium intake. Researchers reported strong positive associations between dietary magnesium intake and indices of skeletal muscle mass in both men and women.

The studies presented are summarized in Table 4.

## 7. Creatine Monohydrate

Creatine is a guanidino compound that is produced endogenously in the liver, pancreas, and kidney from arginine, glycine, and methionine [10], and is also principally found in muscle meats, including fish [88]. Fish such as tuna and salmon are relatively high in creatine [10]. The creatine kinase enzyme phosphorylates creatine in muscle cells to a high-energy molecule, phosphocreatine, which is used as an immediate source of energy in the muscle cells. More recently, there has been an increase in the use of creatine supplementation among young people and sports athletes. Nevertheless, high doses of creatine may lead to cytotoxicity by formaldehyde production [89]. It is well documented that aging is associated with decreased levels of intramuscular creatine [90]. For instance, Candow et al. [91] demonstrated that 10 weeks of creatine supplementation (0.1 g/kg/day) combined with a supervised resistance training program (three days/week) reduces muscle protein degradation and bone resorption in aging males compared to placebo. The researchers concluded that creatine does not result in cytotoxicity (Table 5).

It is important to underline, however, that creatine plays a more important role if associated with physical activity. Chilibeck′s meta-analysis [92] shows that creatine supplementation during resistance training is more effective in increasing lean muscle mass (especially in the upper limbs). It seems, in fact, that creatine improves energy and glycogen stores by increasing endurance, but the mechanisms by which this occurs are still being studied.

## 8. L-Carnitine and Muscle Mass

L-Carnitine (3-hydroxy-4-N-trimethylaminobutyrate) is the active form of carnitine derived from the essential amino acids lysine and methionine, and occurs naturally in a variety of foods of animal origin. Omnivores have a carnitine dietary intake ranging from 20 to 300 mg/day mainly from red meat, fish, and dairy products consumption, while vegetarians consume 1–3 mg/day [93]. The main role of L-carnitine is the transport of long-chain fatty acids across the inner mitochondrial membrane to be oxidized to produce ATP [94] in addition to its anti-inflammatory properties [95].

Recently, it has been proposed that carnitine has a protective effect on muscle mass. These studies are summarized in Table 6. For instance, Sawicka et al. [96] demonstrated that 24 weeks of 1500 mg l-carnitine-l-tartrate supplementation is associated with a better preservation of lean mass in healthy aged women. L-carnitine′s ability to help preserve muscle mass was also confirmed in a randomized controlled clinical trial including 70 centenarians with weakness, decreasing mental health, impaired mobility, and poor endurance [97]. The researchers of that study found that patients taking 2 g of L-carnitine daily for six days had a reduction in fat mass and an increase in lean mass.

## 9. Discussion

Fish is an optimal food for maintaining muscle mass and preventing sarcopenia as it contains a complex array of macro- and micronutrients essential for the proper functioning of the skeletal muscle. In fact, fish is a rich source of PUFAs that are able to reduce inflammation status by improving muscle response to exercise and diet. Frequent consumption of fish appears to increase muscle strength and volume. The Robinson study [42] demonstrated that each additional portion of fatty fish consumed per week was associated with a gain in grip strength, both in men and women. Moreover, with the help of vitamin E introduced into the feed, the anti-inflammatory effect of PUFAs is strengthened, reducing the risk of oxidation of fatty acids. In addition, besides shuttling long-chain fatty acids into the mitochondria for subsequent β-oxidation, L-carnitine exerts an anti-inflammatory effect in several disease settings, thus making fish a fundamental food for human health [96,97]. The high biological value of fish proteins is essential for nourishing muscle mass and meeting the RDA of protein. Indeed, fish proteins have been shown to increase the weight of muscle mass by downregulating the atrophy-related genes. In addition, fish also provides creatine, a substrate for the formation of ATP during intense exercise. The consumption of fish contributes to increasing the intramuscular level of creatine, which has decreased [90].

Many investigations, as highlighted by the meta-analysis by Lehmann et al. [69], indicate that fish consumption contributes to total vitamin D intake. Vitamin D is involved in neuromuscular function and is essential for maintaining calcium and phosphate homeostasis. Data from the same meta-analysis showed that vitamin D levels increased when fish was consumed at least twice a week for four weeks in a row.

Within the complex muscular balance, magnesium, the cofactor of many enzymatic reactions, plays a fundamental role in maintaining muscle mass and protecting it from oxidative damage that can cause structural damage to muscle tissue.

Not everyone consumes fish and some people are vegetarian or vegan by choice. While magnesium is easily introduced through a large consumption of vegetables, for these people, excellent sources of polyunsaturated fatty acids and vitamin E are nuts, plant seeds (e.g., flax seeds and chia seeds), [98] and plant oils [99], while vegetable proteins are easily found in legumes (0.8 g·kg^−1^·day^−1^ recommended for most non-active adults, 1.6–1.7 g·kg^−1^·day^−1^ for strength and power athletes, and 1.2–1.4 g·kg^−1^·day^−1^ for endurance-sport athletes) and also plant seeds [100]. Dietary intakes of vitamin D are low in vegans who do not achieve sufficient sun exposure [101]. Supplements composed of fungal algae and therefore suitable for vegans have recently been produced [98]. In the USA, the IOM recommend an RDA of 600 I.U·day^−1^ [102] for vitamin D. Similarly, the vegan, especially if they are an athlete, must integrate creatine, which has beneficial effects on its performance. The dosage depends on the physical activity practiced [103].

Despite the lack of studies on fish consumption and sarcopenia, fish intake undoubtedly plays an important role in maintaining muscle mass and it is recommended that people eat fish (preferably fatty fish) at least three times a week.

The main limitation of this study is that we have considered single nutrients typical of fish and not fish itself, so the role of a single nutrient does not automatically apply to consumption of a food with a high content of the nutrient.

Finally, it is important to highlight that proper nutrition must be accompanied by adequate physical activity; in fact, although life expectancy has increased mainly thanks to new knowledge in the field of medicine, morbidity caused by excessive sedentary lifestyle is one of the plagues of our time. Inactivity, in fact, has a serious negative effect on the health of the individual [104].

## Figures and Tables

**Table 1:** Summary of studies of omega-3 fatty acids considered in this narrative review.

Authors/Title/Year	Type of Study	Oil Integration	Sample	Duration	Results
Smith et al. 2015	Randomized study	n-3 PUFA (1.86 EPA and 1.50 g DHA/die) or corn oil	Sixty healthy 60–85-year-old men and women	Six months	Treatment with fish oil derived n-3 PUFA brought benefits on thigh muscle volume, handgrip strength, and tended to increase the average isokinetic leg muscle power
Yoshino et al. 2016	Ten subjects of Smith study (2015) with best answer to treatment	n-3 PUFA (1.86 EPA and 1.50 gDHA/die) or corn oil	Twenty healthy 60–85-year-old men and women	Six months	Different pathways involved in mitochondrial regulation and in the organization of the cell matrix were increased while the pathways related to calpain- and ubiquitin-mediated proteolysis, mRNA translation, and inhibition of mTOR signaling were decreased
Rodacki et al. 2012	Randomized study	3 groups: Group ST (only strength-training), Group ST90 (2 g/die fish oil for 90 days) and ST150 (2 g/die fish oil for 150 days)	Forty-five women	90 or 150 days	Supplementation with fish oil accompanied by strength training increased the neuromuscular system, enhancing the muscle strength and the functional capacity in elderly women
Boit et al. 2017	Randomized study	3 g of fish oil	Fifty men and women	18 weeks	long-chain n-3 PUFA supplementation enhances the increases in maximal isometric torque and muscle quality after 18 weeks of resistance exercise in older women but not in older men
Smith et al. 2011	Randomized study	n-3 PUFA (1.86 EPA and 1.50 g DHA/die)	Sixteen healthy adults	8 weeks	Omega 3 PUFA enhance muscle protein synthesis in response to stimulated feeding
McGlory et al. 2016	Randomized study	Fish oil group (5 g/day fish oil) and CONTROL GOUP (5 g/day coconut oil) took proteins following resistance exercise.	Twenty healthy males	8 weeks	No changes were found neither at REST, nor after physical activity (FED) nor after physical activity + protein intake (FEDEX)
Pahor et al. 2018	Pilot randomized clinical trial	0.7 of fish contained 400 mg of EPA and 200 mg of DHA	289 participants with reduced mobility	12 months	found no significant changes on IL-6 or walking speed
Kamolrat et al. 2013	Randomized study	Two group: fish oil (EPA 49.6%, DHA 50.4%) and control oil (60% olive and 40 & soy)	32 male Rowett strain Lister Hooded rats	8 weeks	The results also indicate an increase in anabolic signalling pathways particularly in type 2 fibres without effects on inflammation processes
Gingras et al. 2007	Randomized study	Two groups: control group (control oil mixture based on 60% cotton seed and 40% virgin olive oils), group LC*n*-3PUFA (4% menhaden oil)	Six growing steers	15 weeks	Long-term enteral the supply of LCn-3PUFA gives greater sensitivity to elimination of insulin-regulated amino acids and glucose and that these answers occur, in part, in the skeleton muscle
Kamolrat et al. 2013	In vitro study	Three group of murine C2C12 cells: control, EPA group, and DHA group	Murine C2C12 murine cell	28 h	The cells treated with EPA had greater protein synthesis and a lower protein breakdown than the controls and cells treated with DHA
Siam M, et al. 2008	Retrospective cohort study	No	2983 men and women aged 59 to 73		Each additional portion of fatty fish consumed per week was associated with a gain in grip strength 0.43 kg in men and 0.48 kg in women.

**Table 2:** Summary of studies on fish protein considered in this narrative review.

	Type of Study	Protein Supplementation	Sample	Duration	Results
Alexandrov Nikita et al. 2018	Cohort Study		31,278 men and 43,355 women of 18–91 years		The proteins of animal origin increase the secretion of carnitine, suggesting their ability to preserve muscle mass.
Drotningsvik A et al. 2019	Double-blind randomized controlled pilot study	One intervention arm (5.2 g of whiting hydrolysate) and one control arm (placebo)	24 nursing home residents (60 years or older)	6 weeks	The study is feasible.

**Table 3:** Summary of studies on vitamin D considered in this narrative review.

	Type of Study	Vitamin D Supplementation	Sample	Duration	Results
Antoneta Granic et al. 2017	Newcastle 85+ study: longitudinal study	No supplementation vitamin D	845	5 years	The lowest 25(OH)D season-specific quartile was associated with a faster rate of muscle strength decline in men 85+ years old
Mee Kyoung Kim et al. 2011	Cross-sectional study	No supplementation vitamin D. Result extracted from study conducted periodically since 1998 to assess the health and nutritional status of the noninstitutionalized civilian population of Korea	1380 men and 1789 women aged 50 years or older	From 1998	Strong inverse association between 25(OH)D level and sarcopenia in older Koreans
Knutsen at al. 2010	Cross sectional descriptive study	No supplementation vitamin D	572	2 months	musculoskeletal pain, headache, fatigue is correlated with deficiency of vitamin D
Bischoff-Ferrari et al. 2004		No supplementation vitamin D	32 patients		Vitamin D receptors in muscle decrease with old age
Marieke B. Snijder et al. 2006	Prospective cohort study	No supplementation vitamin D	1231 men and women (aged 65 years and older) Amsterdam	1 year	Low 25(OH)D (10 ng/mL) was associated with an increased risk of falling
Bischoff-Ferrari et al. 2016	Double-blind randomized clinical trial	Three study groups with monthly treatments: 1. Control group receiving 24,000 IU of vitamin D3 (24,000 IU group), 2. 60,000 group receiving 60,000 IU of vitamin D3 3. 24,000 IU plus calcifediol group receiving 24,000 IU of vitamin D3 plus 300 μg of Calcifediol	200 men and women (aged 70 years and older)	1 year	Although higher monthly doses of vitamin D were effective in reaching a threshold of at least 30 ng/mL of 25-hydroxyvitamin D, they had no benefit on lower extremity function and were associated with increased risk of falls compared with 24,000 IU
J.H. Oh et al. 2009	Two group: 1: rotary cuff tear 2. different shoulder	No supplementation vitamin D	366 patients	16 months	Serum vitamin level had a significant negative correlation with fatty degeneration of the torn cuff muscles and a positive correlation with isokinetic muscle performance

**Table 4:** Summary of studies on magnesium considered in this narrative review.

Authors/Title/Year	Type of Study	Magnesium Intake Measurement	Concentration Serum Magnesium Measurement	Sample	Duration	Results
Domingeuez Ligia J et al. 2006	Prospective epidemiologic study	No	Yes	1453	20 months	Higher levels of circulating magnesium correspond to better muscle performance
Welch Alisa A et al. 2016	Cross-sectional study	Yes	No	2570 women aged 18 to 79 years	Data extrapolated over the years from an ongoing study on the aging of healthy adult twins	High intake of magnesium is significantly associated with a higher percentage of Fat Free Mass (FFM), leg explosive power and with circulating CRP concentrations.
Welch Alisa A et al. 2017	Prospective cohort study	Yes	No	502,655 people aged 37–73 years	2006–2010	A greater magnesium intake was related to grip strength and a higher skeletal muscle mass.
Hayhoe Richard P.G. et al. 2019	cross-sectional cohort study	Yes	Yes	25,639 men and women aged 40–79 years old	1993–2000	A higher intake of magnesium and a higher serum magnesium concentration corresponded to better skeletal muscle mass indices in both men and women

**Table 5:** Creatine monohydrate.

Authors/Title/Year	Type of Study	Supplementation Creatine	Sample	Duration	Results
Darren G. Candow. 2008	Randomized double-blind study	Yes	30	10 weeks	Low-dose creatine supplementation enhances lean mass end grip strength but did not lead to formaldehyde production.

**Table 6:** Summary of studies on l-carnitine considered in this narrative review.

Authors/Title/Year	Type of Study	L-Carnitine Supplementation	Sample	Duration	Results
Sawicka Angelika K et al. 2018	Randomized study	Yes 1500 mg once a day	28	24 weeks	Evident better preservation of lean mass in those taking L-carnitine.
Malaguarnera M. 2007	Randomized, double-blind, placebo-controlled study.	Yes 2 g once a day	66 centenarians	6 months	Patients taking L-carnitine had a reduction in fat mass and an increase in lean mass.
